# Assessment of Useful Alien Plant Species Cultivated and Managed in Rural Home Gardens of Limpopo Province, South Africa

**DOI:** 10.1155/2020/3561306

**Published:** 2020-04-20

**Authors:** Sebua Silas Semenya, Alfred Maroyi

**Affiliations:** ^1^Technology Transfer Office, Research Administration and Development Department, University of Limpopo, Private Bag X1106, Sovenga 0727, Limpopo, South Africa; ^2^Department of Biodiversity, University of Limpopo, Private Bag X1106, Sovenga 0727, Limpopo, South Africa

## Abstract

Several communities in developing countries derive substantial part of their livelihood needs from alien plants cultivated and managed in home gardens. The aim of this study was to assess useful alien plant species cultivated and managed in home gardens of Limpopo province in South Africa. Semistructured interviews, personal observation, and guided walks with 300 participants between January 2015 and December 2016 were employed to obtain data on names of alien plants cultivated in home gardens and their use categories. A total of 101 plant species belonging to 44 families were recorded from the study area. More than half of the species (66.3%) belonged to 14 families, Fabaceae, Asteraceae, Rosaceae, Solanaceae, Lamiaceae, Anacardiaceae, Poaceae, Amaranthaceae, Apocynaceae, Brassicaceae, Cactaceae, Euphorbiaceae, Moraceae, and Myrtaceae. Twenty-six use categories of alien plants were identified in this study with the majority of species (75.2%) used for medicinal purposes, followed by ornamental (33.7%), edible fruits (24.8%), spices (16.8%), vegetables (16.8%), shade (11.9%), beverages (10.9%), construction materials (8.9%), firewood (7.9%), and hedge (7.9%). These findings corroborate the existing body of knowledge emphasizing the importance of plants grown and managed in home gardens to the livelihood needs of local communities.

## 1. Introduction

Plant species are an integral part of rural livelihoods in several rural communities across the African continent. Research by Cunningham [1] revealed that plant resources are important in the provision of construction poles, sources of food, medicine, shelter, building materials, fuel, and cash income. Similar research conducted in Cameroon [2, 3], Lesotho [4], Nigeria [5], and South Africa [6] revealed that a significant number of these species required for livelihoods needs are exotic plant species. These are species which have been introduced either intentionally or unintentionally into a country. Exotic species are usually divided into three categories, namely casual, naturalized, and invasive aliens [7, 8]. According to Pyšek et al. [8], casual aliens reproduce occasionally outside cultivation, do not form self-sustaining populations, and rely on repeated introductions for their persistence. Naturalized species are defined as aliens that reproduce consistently without direct human intervention, while invasive aliens are defined as naturalized species producing offspring in large numbers and at considerable distances from the parent plants with the potential to spread over a large area. Many conservation practitioners and scientists regard the effects of invasive alien species in a largely negative light and advocate for scientists and government institutions to research and manage them, with huge budgets set aside for their detection and control [9, 10]. The harmful ecological effects of alien plant species on natural ecosystems, economy, and human health are well-documented with Shackleton and Shackleton [11] arguing that there is need to evaluate the positive and negative impacts of alien plant species on biodiversity, humankind, and economy. Some researchers also argue that several invasive species, especially those introduced purposefully, usually offer economic and intrinsic benefits, which can result in contentious issues and conflicts of interest surrounding their management, since some people may oppose certain forms or methods of control and want to derive benefits from these species [12–18]. Despite increasing evidence which show economic and intrinsic benefits of exotic plants, very few studies have documented such beneficial effects associated with such species. Most studies documenting the economic and intrinsic benefits of exotic plants appear to emphasize the medicinal value of these species. For example, Maroyi [19] documented the therapeutic value of alien plants in Zimbabwe and found that 26 species are used as herbal medicines for both human and animal ailments and diseases. A similar study conducted by Njoroge et al. [20] in Kenya reported 75 species from 34 plant families used as sources of traditional medicines against 59 human ailments. Borokini and Babalola [21] reviewed the utilization potential of eight exotic plant species used by Nigerians, and found that these species play a crucial role as sources of charcoal and herbal medicines. Several studies in Eastern Cape and Limpopo provinces in South Africa [22–29] focused on exotic plants used as herbal medicines in the country. Therefore, the present study was aimed at investigating the use values of alien plants cultivated and managed in the home gardens in Limpopo province of South Africa.

## 2. Methodology

### 2.1. Study Area

**Figure 1:**
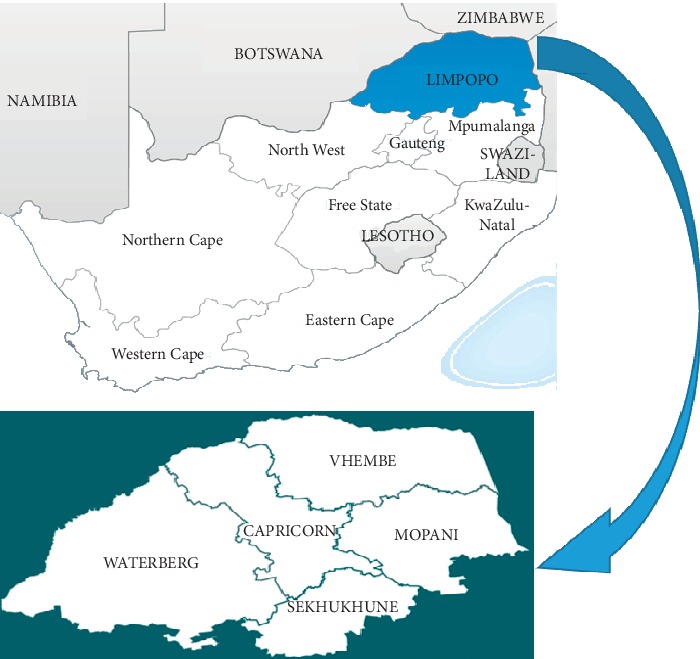
Map of Limpopo province indicating the studied areas.

The present study was conducted in five districts namely Capricorn, Mopani, Sekhukhune, Waterberg, and Vhembe which constitute Limpopo province of South Africa (Figure 1). The Bapedi-speaking people constitute the largest cultural group in the province constituting 57.0% of the population [30]. Limpopo province receives mean annual rainfall ranging from 200 mm to 1500 mm and mean annual temperature ranging from 8°C to 32°C [31]. The major vegetation types in Limpopo province include bushveld, grassland, and forest biomes [32].

### 2.2. Data Collection

A multipurpose household survey was conducted from January 2015 to December 2016 with the aim of (i) randomly selecting the home gardens, (ii) informing the owners of chosen home gardens about the aim of the study, and (iii) requesting consent from informants to participate in data gathering and collection of voucher specimens. A total of 300 home gardens were assessed, resulting in 60 home gardens sampled per district. Information collected via semistructured interviews included sociodemographic characteristics of the participants, diversity of useful alien plant species, and how these species are used. The researchers collected, pressed, dried, and deposited voucher specimens at the University of Limpopo's Herbarium for documentation and reference purposes.

### 2.3. Data Analysis

Analyses of useful alien plant species were conducted using Statistical Package for the Social Sciences (SPSS) and Palaeontological Statistics [33]. The local importance of alien plant species was assessed using the relative frequency of citation (RFC). This index, proposed by Tardío and Pardo-de-Santayana [34], shows the local importance of each species, and it is a result of the frequency of citation (FC), the number of informants mentioning the use of the species divided by the total number of informants (*N*), without considering the use categories [34, 35]. This index varies from 0, where nobody refers to the plant as useful, to 1 in the likely case that all the informants would mention the use of the species.

(1) $$\begin{array}{c} \text{RCN}=\frac{\text{FC}}{N}. \end{array}$$

## 3. Results and Discussion

### 3.1. Sociodemographic Characteristics of the Participants

Table 1 shows the demographic characteristics of the participants. The majority of participants belonged to the Bapedi ethnic group (60.0%), and the remainder were Vhavenda and Vatsonga (20.0% each). The majority of participants were females (73.3%) compared to 26.7% males. Their ages ranged from 20 years to 77 years, with 38 years as the median. The majority (63.3%) were between 31 years and 40 years of age; 16.6% were between 51 years and 60 years; 11.0%, aged between 41 years and 50 years; 6.3%, aged between 20 years and 30 years; and 2.6% were above 70 years of age. Majority of the participants were married with 53.7% of the men married, while 70.4% of the women were married. About three quarters of the participants (71.7%) were educated up to secondary school level followed by 11.3% who were educated up to primary school level, 9.7% had postsecondary qualifications, and 7.3% did not attend schooling. The main economic activity of the participants in this study was agriculture (48.0%), followed by shop keeping (43.0%), crafting (6.0%), and livestock herding (3.0%).

### 3.2. Plant Use and Taxonomic Diversity

**Figure 2:**
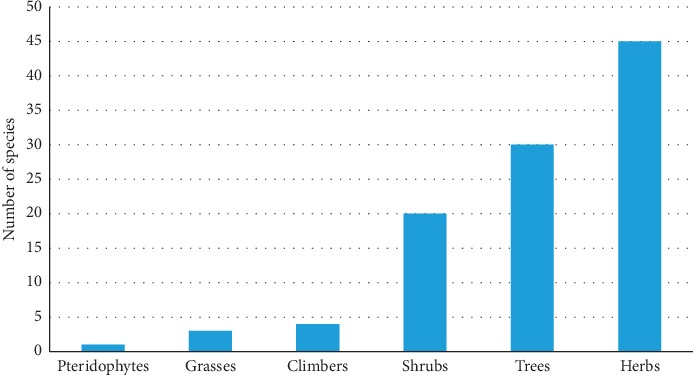
Growth forms of alien plant species recorded in rural home gardens of Limpopo province.

A total of 101 plant species were recorded (Table 2) with herbs, trees, and shrubs having the most species (Figure 2). Pteridophytes and gymnosperms were represented by a single species each, *Nephrolepis exaltata* (L.) Schott (family Nephrolepidaceae) and *Pinus patula* Shiede ex SchItdl & Cham (family Pinaceae). A large number (66.3%) of the plant species recorded are from 14 families (Table 3), and the other 30 families had less representation, between one and two species each. Plant families with the highest number of species were Fabaceae (9 species), Asteraceae (8 species), Rosaceae and Solanaceae (7 species each), Lamiaceae (6 species), Anacardiaceae (5 species), Poaceae (4 species), and Amaranthaceae, Apocynaceae, Brassicaceae, Cactaceae, Euphorbiaceae, Moraceae, and Myrtaceae (3 species each) (Table 3). All these plant families with the exception of Moraceae are among the largest plant families in the world, characterized by at least 2000 species each [36].

### 3.3. Plant Use Categories Based on Relative Frequency Citation (RFC) Values

**Figure 3:**
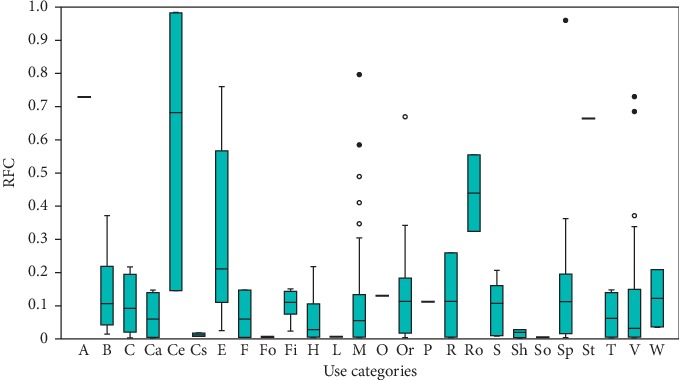
Relationship of relative frequency citation (RFC) and use categories of alien plants in home gardens in Limpopo province, South Africa. Conventions: A = facilitate divination, B = beverage, C = construction materials, Ca = carvings, Ce = edible grain and cereals, Cs = cosmetics, E = edible fruits, F = fibre, Fo = fodder, Fi = firewood, H = hedge, L = lubricant, M = medicines, O = oil, Or = ornamentals, P = perfume, R = recreational, Ro = edible roots, S = shade, Sh = shampoo, So = soap, Sp = spice, St = edible stems, T = tool handles, V = vegetables, and W = windbreak.

**Figure 4:**
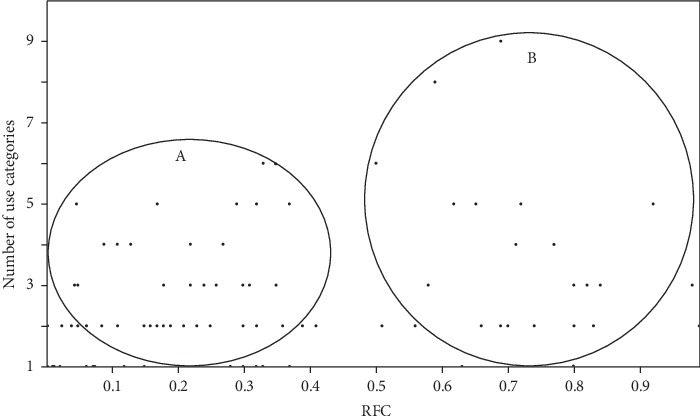
Relationship between the number of use categories and relative frequency citation (RFC) of alien plants in home gardens in Limpopo province, South Africa.

Twenty-six use categories of alien plants were identified in this study (Table 2, Figure 3). Relative frequency citation values determined in this study indicated that alien species used to facilitate divination and other spiritual healing rituals (A, RFC = 0.73) and used as edible grain and cereals (Ce, RFC = 0.15–0.99), edible fruits (E, RFC = 0.04–0.76), roots (Ro, RFC = 0.32–0.55), and stems (St, RFC = 0.66) were characterized by the highest values (Figure 3). The RFC shows the importance of each species based on the number of participants citing the species [35]. The RFC values of different species' use categories were summed resulting in a numerical value that was used to rank the species in order of importance displayed in Table 2. The species with RFC values ≥0.8 were *Zea mays* L. (cereal and herbal medicine), *Allium cepa* L. (herbal medicine and spice), *Opuntia ficus-indica* Mill. (beverage, edible fruits, herbal medicine, ornamental, and edible roots), *Punica granatum* L. (beverage, edible fruits, and herbal medicines), *Moringa oleifera* Lam. (herbal medicine and vegetable), *Solanum lycopersicon* L. (edible fruits, herbal medicine, and vegetable), *Catharanthus roseus* (L.) G.Don. (herbal medicine), *Musa paradisiaca* L. (beverage, edible fruits, and herbal medicine), and *Schkuhria pinnata* (Lam.) Kuntze ex Thell (herbal medicine and vegetable) (Table 2). Assessment of RFC values in relation to use categories resulted in grouping plant species recorded in this study into two clusters, A and B, as shown in Figure 4. Cluster A is composed of species characterized by RFC values which are below 0.5 and use values which are less than six while cluster B is composed of species with RFC values which are higher than 0.5 and number of use values which are as high as nine (Figure 4). Multipurpose species within cluster B characterized by at least three use categories and RFC values ≥0.5 included *Allium cepa* L., *Opuntia ficus-indica* Mill., *Punica granatum* L., *Musa paradisiaca* L., *Ricinus communis* L., *Citrus lemon* (L.), Burm. f., *Cannabis sativa* L., *Schinus molle* L., *Agave americana* L., *Carica papaya* L., *Jacaranda mimosifolia* D. Don., *Psidium guajava* L., and *Morus alba* L. (Table 2).

**Figure 5:**
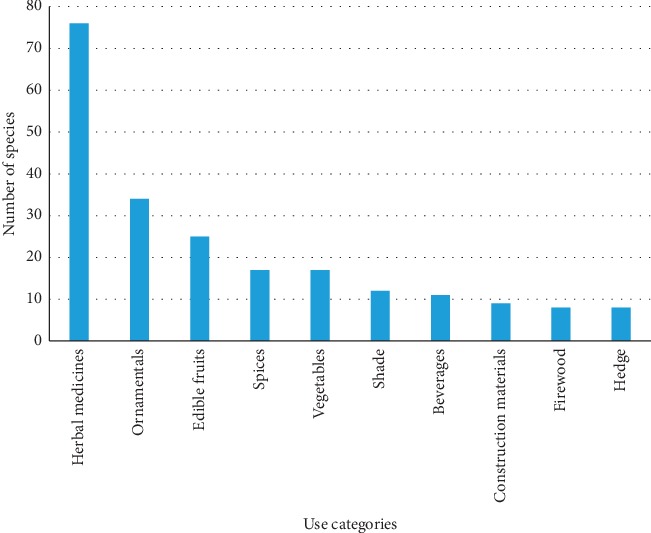
Major use categories of exotic plants in home gardens in Limpopo province.

The majority of plant species recorded in this study (75.2%) were used for medicinal purposes, followed by ornamental plants (33.7%), edible fruits (24.8%), spices (16.8%), vegetables (16.8%), shade (11.9%), beverages (10.9%), construction materials (8.9%), firewood (7.9%), and hedge (7.9%) (Figure 5). These results correlate with previous research findings from South Africa [37, 38] and other countries like Brazil [39], Iberian Peninsula [40], and India [41] which found that the majority of exotic plant species in the home gardens are used as traditional medicines. High usage of species for medicinal purposes in the Limpopo province is not surprising as the province is characterized by inadequate modern health care services and shortage of pharmaceutical drugs for different ailments. Recent research by Ntuli and Maboya [42] showed that there is a shortage of medical doctors and maladministration of public sector hospitals in the rural areas of the Limpopo province, with the majority of medical doctors employed in urban areas. There is now overwhelming evidence that alien plant species are used widely as herbal medicines and are now recognized as an important component of indigenous pharmacopoeia in several countries [43–46]. Research by Palmer [44] and Alencar et al. [45, 46] revealed that utilization of alien plants as herbal medicines is a result of experimentations conducted for several years and represents an adaptation of this culture over time. Therefore, alien plants are included in traditional pharmacopoeias to fill therapeutic vacancies that native plants cannot satisfy [43–46].

The recorded food plants were used as beverage, edible grain and cereals, edible fruits, roots, stems, and spices (Table 2). The majority (40.3%) of recorded food plants were consumed raw and these included *Anacardium occidentale* L.*, Carica papaya*, *Citrus lemon*, *Eriobotrya japonica* L., *Ficus carica* L., *Ficus platypoda* (Miq.) A.Cunn. ex Miq., *Morus alba* L., *Musa paradisiaca* L., *Opuntia ficus-indica*, *Persea americana* Mill.*, Prunus persica* (L.) Batch var. persica*, Psidium guajava* L., *Punica granatum* L., *Rhus succedanea* L.*, Rubus cuneifolius* Pursh, and *Schinus molle* L. Some species which included *Hylocereus undatus* (Haw.) Britt. & Rose, *Litchi chinensis* Sonn., *Mangifera indica* L., *Passiflora edulis* Sims, *Prunus armeniaca* L., *Prunus persica*, *Rubus ellipticus* Pursh, and *Vitis vinifera* L. were managed in home gardens for their edible fruits. About a third of the documented species (29.0%) were consumed as vegetables and these included *Acanthus montanus* L., *Alternanthera pungens* Kunth, *Bidens pilosa* L.*, Cannabis sativa* L., *Datura ferox* L., *Schkuhria pinnata* (Lam.) Kuntze ex Tehell, *Beta vulgaris* L., *Ficus platypoda, Solanum lycopersicum* L., *Brassica juncea* (L.) Czern, *Brassica oleracea* L., *Brassica rapa* L., *Carica papaya*, *Jatropha curcas* L., *Solanum lycopersicum*, *Moringa oleifera* Lam., and *Morus alba*. The vegetables managed in home gardens were eaten as relish to complement staple diet prepared from maize meal (*Zea mays* L.). The plants species used as spices (20.9%) included *Allium sativum* L., *Lavandula angustifolia* Mill., *Mentha spicata* L., *Aframomum melegueta* (Rox.) K.Schum., *Capsicum Chinese* L., *Coriandrum sativum* L., *Melia azedarach* L., *Mentha longifolia* L., *Ocimum basillium* L., *Rosmarinus officinalis* L., *Ruta graveolens* L., and *Zingiber officinale* Rosc. The remaining species which constituted the food category in the present study were consumed as beverages and these included *Artemisia annua* L., *Mentha longifolia*, and *Zingiber officinale*, edible grain and cereals (*Sorghum bicolor* (L.) Moench., *Vigna unguiculata* (L.) Walp., and *Zea mays*), edible tubers (*Daucus carota* L. and *Ipomoea batatas* (L.) Lam.), and edible stems (*Saccharum officinarum* L.) and used as cooking oil (*Helianthus annuus* L.). Research by Gosh [47] showed that local food production in the home gardens could meaningfully contribute towards building a sustainable food production for the household and local community. Similarly, Kamiyama et al. [48] argued that there is need to strengthen food production in local communities through improved production in home gardens to mitigate growing global food instability.

The proportion of ornamental plants was about a third (33.6%) to the overall use categories of the home garden alien flora in Limpopo province. The majority of these species were categorized as multipurpose species with *Campuloclinium macrocephalum* (Less.) DC., *Canna indica* L., *Cereus jamacaru* DC., *Mentha spicata*, *Monstera deliciosa* Liebm, and *Spathodea campanulata* P.Beauv. exclusively cultivated and managed as ornamental plants (Table 2). The relevance of ornamental plants in home gardens varies in different countries. Research by Aworinde et al. [49] revealed that food and medicinal plants were more abundant than ornamental plants but research findings of Neulinger et al. [50] revealed the opposite, with ornamental plants exceeding both food and medicinal plants. Therefore, the importance of ornamental plants in home gardens should not be underestimated and Hurrel [51] argued that ornamentals have been employed by humans throughout history for aesthetic and symbolic values. Similarly, only nine species which included *Acacia dealbata* Link, *Acacia mearnsii* De Wild, *Agave americana* L., *Eucalyptus camaldulensis* Dehnh., *Eucalyptus paniculata* Sm., *Jacaranda mimosifolia* D. Don., *Pinus patula* Shiede ex SchItdl and Cham, *Schinus molle* L., and *Sesbania punicea* (Cav.) Benth. were used as sources of timber and construction materials (Table 2). Plant species such as Acacia *dealbata*, *Acacia mearnsii*, *Agave Americana, Eucalyptus camaldulensis*, *Eucalyptus paniculata*, and *Jacaranda mimosifolia* are widely cultivated in other countries such as Ethiopia, Kenya, and Mexico as sources of timber and construction materials [52–54]. Species used as sources of firewood included *Acacia dealbata*, *Acacia mearnsii*, *Eucalyptus camaldulensis*, *Eucalyptus paniculata*, *Jacaranda mimosifolia*, Jatropha curcas, and *Schinus molle* (Table 2).

## 4. Conclusions

Due to high diversity of alien species cultivated and managed in home gardens in Limpopo province and the associated wide range of use categories of these species, it implies that home gardens could be important sources of goods and ecosystem services needed by local communities. Results of this study corroborate the existing body of knowledge emphasizing the importance of plants that are grown and managed in home gardens. Exploitation of plants cultivated and maintained in home gardens has played an important role in the provision of livelihood needs of local communities. Future research needs to quantify the goods and ecosystem services provided by alien plants. Such detailed studies are needed to understand the importance of alien species in the provision of livelihood needs. We conclude that future management polices focusing on alien plants should take into consideration the positive attributes of such plant species.

## Figures and Tables

**Table 1:** Socioeconomic characteristics of the study sample, *N* = 300.

Socioeconomic variables		Number	%
Ethnic groups	Bapedi	180	60.0
	Vhavenda	60	20.0
	Vatsonga	60	20.0

Gender	Female	220	73.3
	Male	80	26.7

Age (years)	<30	12	4.0
	21–30	19	6.3
	31–40	170	56.7
	41–50	46	15.3
	51–60	32	10.7
	61–70	13	4.3
	>70	8	2.7

Marital status	Single (males)	22	7.3
	Single (females)	41	13.7
	Married (males)	43	14.3
	Married (females)	155	51.7
	Divorced or separated (males)	15	5.0
	Divorced or separated (females)	24	8.0

Highest level of education	No education	22	7.3
	Primary	34	11.3
	Secondary	215	71.7
	Tertiary	29	9.7

Economic activity	Agriculture	144	48.0
	Shop keeping	129	43.0
	Crafting	18	6.0
	Livestock herding	9	3.0

**Table 2:** Alien plant species recorded in rural home gardens of the Limpopo province.

Botanical and family name	Habit	Use categories^*∗*^																										RFC
		A	B	C	Ca	Ce	Cs	E	F	Fo	Fi	H	L	M	O	Or	P	R	Ro	S	Sh	So	Sp	St	T	V	W	
*Zea mays* L., Poaceae	Grass					X								X														0.99
*Allium cepa* L., Amaryllidaceae	Herb													X					X				X					0.98
*Opuntia ficus-indica* Mill., Cactaceae	Shrub		X					X						X		X			X									0.92
*Punica granatum* L., Lythraceae	Tree		X					X						X														0.84
*Moringa oleifera* Lam., Moringaceae	Shrub													X												X		0.83
*Solanum lycopersicum* L., Solanaceae	Herb							X						X												X		0.82
*Catharanthus roseus* (L.) G.Don., Apocynaceae	Herb													X														0.80
*Musa paradisiaca* L., Musaceae	Herb		X					X						X														0.80
*Schkuhria pinnata* (Lam.) Kuntze ex Thell, Asteraceae	Herb													X												X		0.80
*Ricinus communis* L., Euphorbiaceae	Herb						X						X	X								X						0.77
*Persea americana* Mill., Lauraceae	Tree							X						X														0.74
*Rhus succedanea* L., Anacardiaceae	Tree							X						X														0.74
*Brassica oleracea* L., Brassicaceae	Herb																									X		0.73
*Citrus lemon* (L.) Burm. f., Rutaceae	Shrub		X					X						X		X				X								0.72
*Cannabis sativa* L., Cannabaceae	Herb													X				X					X			X		0.71
*Vigna unguiculata* (L.) Walp., Fabaceae	Herb					X								X														0.70
*Alternanthera pungens* Kunth, Amaranthaceae	Herb													X												X		0.69
*Schinus molle* L., Anacardiaceae	Tree		X	X				X			X	X		X		X							X				X	0.69
*Saccharum officinarum* L., Poaceae	Grass													X										X				0.66
*Agave americana* L., Agavaceae	Shrub			X					X			X		X		X												0.65
*Acorus calamus* L., Acoraceae	Herb													X														0.63
*Carica papaya* L., Caricaceae	Tree		X					X						X							X					X		0.62
*Jacaranda mimosifolia* D. Don., Bignoniaceae	Tree			X							X	X		X		X		X		X							X	0.59
*Psidium guajava* L., Myrtaceae	Tree		X					X						X														0.58
*Beta vulgaris* L., Amaranthaceae	Herb													X												X		0.56
*Plumeria obtusa* L., Apocynaceae	Tree															X	X											0.51
*Morus alba* L., Moraceae	Tree		X					X						X		X				X						X		0.50
*Bidens pilosa* L., Asteraceae	Herb													X												X		0.41
*Senna occidentalis* (L.) Link, Fabaceae	Tree													X		X												0.39
*Daucus carota* L., Apiaceae	Herb																		X									0.37
*Eriobotrya japonica* (Thunb.) lindl., Rosaceae	Tree							X						X		X				X			X					0.37
*Allium sativum* L., *Amaryllidaceae*	Herb																		X				X					0.36
*Eucalyptus camaldulensis* Dehnh., Myrtaceae	Tree			X	X						X			X		X				X								0.35
*Zingiber officinale* rosc., Zingiberaceae	Herb		X											X									X					0.35
*Eucalyptus paniculata* sm., Myrtaceae	Tree			X							X			X		X				X					X			0.33
*Mangifera indica* L., Anacardiaceae	Tree							X																				0.33
*Monstera Deliciosa Liebm*, Araceae	Shrub															X												0.33
*Ficus carica* L., Moraceae	Tree							X						X														0.32
*Jatropha curcas* L., Euphorbiaceae	Shrub				X						X	X		X												X		0.32
*Ipomoea batatas* (L.) Lam., Convolvulaceae	Climber																		X									0.32
*Mentha longifolia* L., Lamiaceae	Herb		X											X									X					0.31
*Ocimum basillium* L., Lamiaceae	Herb													X									X					0.3
*Rubus cuneifolius* Pursh, Rosaceae	Shrub							X						X									X					0.30
*Solanum mauritianum* scop., Solanaceae	Herb													X														0.30
*Xanthium strumarium* L., Asteraceae	Herb													X		X												0.30
*Prunus persica* (L.) batsch var. *persica*, Rosaceae	Tree		X					X						X		X				X								0.29
*Artemisia vulgaris* L., Asteraceae	Herb													X														0.28
*Senna didymobotrya* (Fresen.) Irwin & Barneby, Fabaceae	Tree											X		X		X				X								0.27
*Acanthus montanus* L., Acanthaceae	Herb													X		X										X		0.26
*Ruta graveolens* L., Rutaceae	Tree													X									X					0.25
*Caesalpinia decapetala* (Roth) Alston., Fabaceae	Shrub											X		X		X												0.24
*Canna indica* L., Cannaceae	Herb															X												0.23
*Lantana camara* L., Verbenaceae	Shrub													X		X												0.23
*Melia azedarach* L., Meliaceae	Tree													X		X				X			X					0.22
*Capsicum Chinese* L., Solanaceae	Herb							X						X									X					0.22
*Prunus armeniaca* L., Rosaceae	Tree							X																				0.22
*Coriandrum sativum* L., Apiaceae	Herb													X									X					0.21
*Kalanchoe tubiflora* (Harv.) Raym-Hamet, Crassulaceae	Herb													X		X												0.19
*Nerium oleander* L., Lamiaceae	Shrub													X		X												0.18
*Nicotiana glauca* Graham, Solanaceae	Shrub	X												X				X										0.18
*Artemisia annua* L., Asteraceae	Herb		X											X														0.17
*Pinus patula* Shiede ex SchItdl & Cham, Pinaceae	Tree			X							X					X				X							X	0.17
*Nephrolepis exaltata* (L.) Schott, Nephrolepidaceae	Herb													X		X												0.16
*Anacardium occidentale* L., Anacardiaceae	Tree							X						X														0.15
*Acacia dealbata* link, Fabaceae	Tree			X							X																	0.15
*Sorghum bicolor* (L.) Moench., Poaceae	Grass					X																						0.15
*Acacia mearnsii* de Wild, Fabaceae	Tree			X							X	X													X			0.13
*Euphorbia prostrata* Aiton, Euphorbiaceae	Herb													X														0.12
*Litchi chinensis* Sonn., Sapindaceae	Tree							X																				0.12
*Brassica juncea* (L.) Czern, Brassicaceae	Herb													X												X		0.11
*Ficus platypoda* (Miq.) A.Cunn. ex miq., Moraceae	Shrub							X	X					X												X		0.11
*Araujia sericifera* Brot., Apocynaceae	Shrub								X				X			X						X						0.09
*Schinus terebinthifolius* Raddi, Anacardiaceae	Tree													X		X												0.086
*Rubus ellipticus* Sm., Rosaceae	Shrub							X																				0.076
*Vitis vinifera* L., Vitaceae	Climber							X																				0.076
*Passiflora edulis* sims, Passifloraceae	Climber							X																				0.073
*Cardiospermum grandiflorum* sw., Sapindaceae	Climber													X														0.063
*Hylocereus undatus* (haw.) Britt. & rose, Cactaceae	Shrub							X																				0.063
*Medicago sativa* L., Fabaceae	Tree									X				X														0.063
*Nymphaea mexicana* Zucc., Nymphaeaceae	Herb													X		X												0.05
*Rosmarinus officinalis* L., Lamiaceae	Herb													X									X					0.05
*Symphytum officinale* L., Boraginaceae	Herb						X							X							X							0.05
*Datura ferox* L., Solanaceae	Herb													X		X										X		0.046
*Sesbania punicea* (Cav.) Benth., Fabaceae	Shrub			X								X		X		X				X								0.046
*Aframomum melegueta* (Rox.) K.Schum., Zingiberaceae	Herb													X									X					0.04
*Spathodea campanulata* P.Beauv., Bignoniaceae	Tree															X				X								0.026
*Brassica rapa* L., Brassicaceae	Herb																									X		0.023
*Campuloclinium macrocephalum* (less.) DC., Asteraceae	Herb															X												0.016
*Urtica dioica* L., Urticaceae	Herb													X														0.016
*Helianthus annuus* L., Asteraceae	Herb														X													0.013
*Argemone ochroleuca* sweet, Papaveraceae	Herb													X														0.01
*Chenopodium album* L., Amaranthaceae	Herb													X														0.003
*Chromolaena odorata* (L.) king & robinson, Asteraceae	Shrub													X														0.003
*Cereus jamacaru* DC, Cactaceae	Shrub															X												0.003
*Lavandula angustifolia* Mill., Lamiaceae	Herb																						X					0.003
*Leucaena leucocephala* (Lam.) de wit., Fabaceae	Tree													X														0.003
*Lolium multiflorum* Lam., Poaceae	Herb													X														0.003
*Pyracantha angustifolia* (franch.) C.K.Schneid., Rosaceae	Shrub													X														0.003
*Sida spinosa* L., Malvaceae	Shrub													X														0.003
*Datura stramonium* L., Solanaceae	Herb													X		X												0.003
*Mentha spicata* L., Lamiaceae	Herb															X							X					0.001

^*∗*^A = divination, B = beverage, C = construction materials, Ca = carvings, Ce = edible grain and cereals, Cs = cosmetics, E = edible fruits, F = fibre, Fo = fodder, Fi = firewood, H = hedge, L = lubricant, M = medicines, O = oil, Or = ornamentals, P = perfume, R = recreational, Ro = edible roots, S = shade, Sh = shampoo, So = soap, Sp = spice, St = edible stems, T = tool handles, V = vegetables, and W = windbreak.

**Table 3:** Plant families with the largest number of alien species (with at least three species) in rural home gardens of Limpopo province.

Family	Number of species	%
Fabaceae	9	8.9
Asteraceae	8	7.9
Rosaceae	7	6.9
Solanaceae	7	6.9
Lamiaceae	6	5.9
Anacardiaceae	5	5.0
Poaceae	4	4.0
Amaranthaceae	3	3.0
Apocynaceae	3	3.0
Brassicaceae	3	3.0
Cactaceae	3	3.0
Euphorbiaceae	3	3.0
Moraceae	3	3.0
Myrtaceae	3	3.0

## Data Availability

All data associated with the manuscript have been included in the tables and figures.
